# An FEM Study on Minimizing Electrostatic Cross-Talk in a Comb Drive Micro Mirror Array

**DOI:** 10.3390/mi15080942

**Published:** 2024-07-24

**Authors:** Andreas Neudert, Peter Duerr, Mario Nitzsche

**Affiliations:** Fraunhofer Institute for Photonic Microsystems IPMS, Maria-Reiche Str. 2, 01109 Dresden, Germany; peter.duerr@ipms.fraunhofer.de (P.D.); mario.nitzsche@ipms.fraunhofer.de (M.N.)

**Keywords:** computer-generated holography, MEMS, phase modulation, comb drive actuator, electrostatic cross-talk, finite element simulation

## Abstract

We are developing a phase-modulating micro mirror-array spatial light modulator to be used for real holography within the EU-funded project REALHOLO, featuring millions of pixels that can be individually positioned in a piston mode at a large frame rate. We found earlier that an electrostatic comb-drive array offers the best performance for the actuators: sufficient yoke forces for fast switching even at low voltages compatible with the CMOS addressing backplane. In our first design, the well-known electrostatic cross-talk issue had already been much smaller than would have been possible for parallel-plate actuators, but it was still larger than the precision requirements for high-image-quality holography. In this paper, we report on our analysis of the crucial regions for the electrostatic cross-talk and ways to reduce it while observing manufacturing constraints as well as avoiding excessively high field strengths that might lead to electrical breakdown. Finally, we present a solution that, in FEM simulations, reduces the remaining cross-talk to well below the required specification limit. This solution can be manufactured without any additional processing steps and suffers only a very small reduction of the yoke forces.

## 1. Introduction

Augmented, virtual, or mixed reality (AR/VR/MR) 3D displays have recently attracted a lot of attention, as can be seen in international conferences on the topic (e.g., [1]) or optics in general. Several systems are already readily available (e.g., [2,3] and many others), but with these, many users experience unwanted physiological side effects like motion sickness. This is a well-known issue in AR/VR/MR displays [4] and is still a field of scientific studies today, e.g., [5]. The effect is mostly due to the accommodation-vergence-conflict, which is especially pronounced in stereo 3D displays with images close to the user. A common mitigation is to limit the scene’s depth content to rather large distances, but this does not work perfectly and, for many applications, is not feasible at all, especially when manual interaction with the virtual ‘objects’ is desired. In addition, a natural visual experience also requires all other depth cues to be presented and coordinated properly.

While there are many research groups trying to improve the stereo image concept to mitigate the accommodation-vergence-conflict in 3D displays in various ways, see [6,7,8,9,10,11,12,13,14], for example, we still believe that the best possible way is to generate fully realistic images by real holography. The possible difficulty in reaching a sufficiently high image resolution is addressed in our project by using a large pixel count for a limited-sized eye-box, as discussed below. Partners within the project are addressing the computational requirements, see [15].

Unfortunately, the term ‘holography’ is widely used for all kinds of displays, often even for 2D imaging. In this paper, we use this term in its original sense only for a display that realistically reconstructs the field of light in three dimensions, just as if it came from a real object. A display, as opposed to a still image, moreover, allows to update the image to show moving scenes.

Holographic displays thus require the spatial and temporal modulation of coherent light. Phase modulation of the light is better suited for holographic displays than amplitude modulation, as discussed in [16]. Very often, the position map for the pixels is computed by iterative algorithms like Gerchberg–Saxton, see [17], which can be quite time-consuming. For real-time applications it is, therefore, advantageous to use phase spatial light modulators as a basis for complex amplitude modulation, see [15].

The huge amount of information in a hologram requires as many millions of individually addressable pixels as possible. To keep the effort at all manageable, it is sufficient to provide only a small ‘viewing window’ for the user’s eyes with image content, e.g., [18]. Even then, the SLM will have quite challenging specifications.

The phase modulation of coherent light could, in principle, be achieved by existing SLMs based on LCoS (liquid crystal on silicon) technology (e.g., [19,20,21,22,23,24,25]). Compared to these, micro mirror arrays (MMAs) can have many advantages for real holography: the phase across a pixel is more uniform, there may be much less cross-talk between neighbor pixels, they can be switched much faster, and they are independent of polarization. Well-known MMAs are offered by Texas Instruments as Digital Light Processing technology (DLP, see [26]). These, however, have been optimized for 2D-image projection and are not well suited for holography: the pixels deflect in tilt mode instead of the piston mode preferred in holography, and there are only two possible addressing states for each pixel instead of the many required here. The few existing piston-mode MMAs (e.g., [27,28,29]) usually have quite large pixels (several ten micrometers and more) and, therefore, much too few pixels for good quality holography. An interesting development at Texas Instruments ([30,31,32]) will have very many pixels of only 10.8 µm pitch, but the actuator can only be addressed to 16 different levels that are quite non-uniformly distributed over the stroke range. While the authors of these papers argue that this is sufficient, we think that some image quality metrics require much better actuator resolution, see [15,33].

So, modulators with the required properties are not available today, but they should be feasible to make with present-day technology, see [34,35,36,37,38,39,40,41,42]. IPMS, together with consortium partners, is currently developing such modulators within the EU H2020 project REALHOLO, see [43]. The work is based on decades of IPMS experience in developing SLMs for other applications and contributions of consortium partners in other required fields like real holographic 3D display concepts, CMOS backplane design and fabrication, packaging, addressing electronics, and demonstrator setup.

As a first application, REALHOLO is aiming at an automotive driver assistance holographic 3D display. For a good-quality holographic image, the project partners anticipate that the SLM needs to fulfill the specifications in Table 1.

The very tight requirement for the deflection precision of 8-bit is especially relevant for this paper. The necessity for this is discussed in [15].

**Figure 1:**
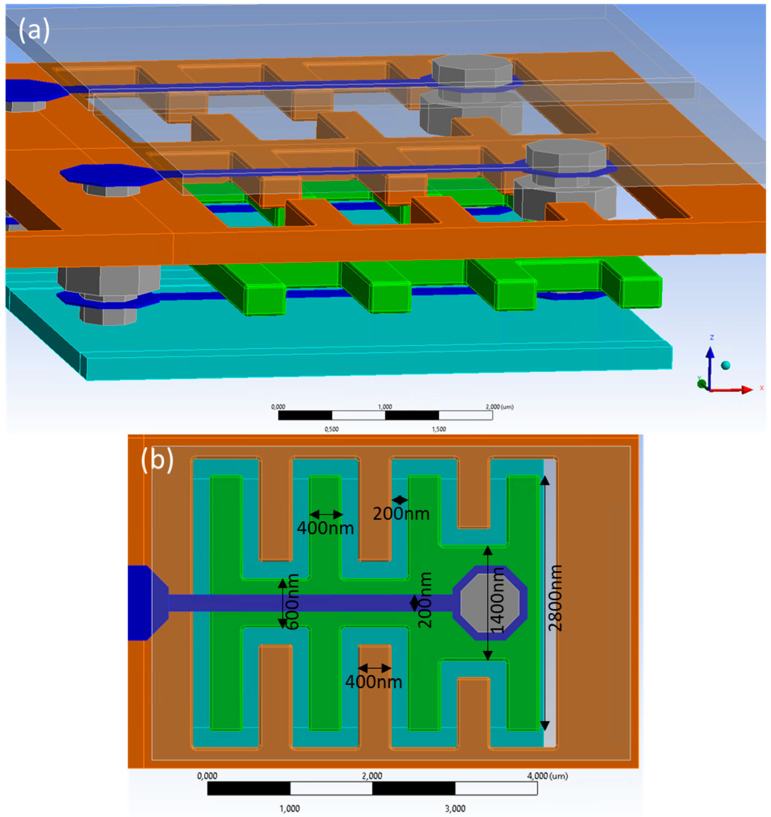
Geometry model of the comb-drive actuator. In (**a**) the geometry of two neighboring pixels is shown. The finger structure of the comb drive is shown in a top-down view in (**b**).

To reach these challenging specifications, we have conceived an electrostatic comb drive actuator, as shown in Figure 1. The development and reasons for the respective choices of the actuator concept are discussed in [34,35,36,37,38]. Due to the small pixels, this design features one-sided hinges, as discussed in [34], but here, we decided to have these in two separate layers, forming a parallelogram-guided actuator for hugely improved robustness and fabrication error tolerance [35,36,37,38]. The MEMS manufacturing technology is described in [39,40]. The first characterization results of manufactured actuators can be found in [41,42]. This paper here, in continuation of the above work, is fully focused on the reduction of the cross-talk between neighbor actuators.

In Figure 1, the two hinges are shown in dark blue. Their thickness is only about 30 nm. While variations of the hinge design are also under investigation within the REALHOLO project, those findings will be published elsewhere. The fixed stator plane is shown in dark orange and the moveable yoke in green. Both of these are 300 nm thick and have a distance (as fabricated) of 300 nm. The baseplate is 330 nm below the yoke and is included in the simulations since it also has an important impact on the electrostatic field distribution. The mirror is 430 nm above the stator and is shown partially transparent for better visibility of the remaining structures.

In a comb drive, the electrostatic fields are strongly concentrated between the comb fingers, which gives rather large forces even for small pixels. At the same time, this field concentration promises a low cross-talk between neighbor pixels, which is needed for the desired high deflection precision. As discussed in [37], this actuator design really has a much smaller cross-talk (0.65%) than a simpler parallel-plate actuator, but it was still larger than the desired value of much less than 0.4%. Furthermore, it is also not prone to a vertical electrostatic pull-in because when moving upwards, the direction of the electric field becomes more and more misaligned with the mirror deflection direction. Only a lateral pull-in could potentially happen in the case of overlay errors between the stator and yoke, but this danger is strongly reduced by the usage of the dual hinge mounting [44].

The moderate cross-talk that we found for this initial design could, in principle, be considered in the calculation of the addressing voltages, but this would require extra effort in this data computation, which is already quite demanding. Therefore, we changed the basic design above systematically and did FEM simulations using Ansys Mechanical 2021 to understand the critical areas of the electrostatic field and find a solution with even less cross-talk.

Other groups also work on minimizing cross-talk in comb drives; see [45,46]. Depending on the general drive layout, different strategies to reduce cross-talk can be applied. In our design, the finger thickness is of the same order of magnitude as the finger width. Therefore, the electric fields are less well confined within the comb drive fingers, and additional means were investigated to reduce the cross-talk even further.

Obviously, the cross-talk could be completely suppressed by a conducting vertical shield wall between all the pixels. However, such a shield wall would have a fixed electrical potential for all pixels and would unavoidably be close to other actuator structures that carry a different electrical potential. If the gap between these elements is very small, the field strength will be very large and electrical breakdown will occur. Leaving a larger gap, on the other hand, reduces the shielding effect, so such shielding structures have to be designed very carefully and still cannot work perfectly.

Depending on the geometry, such a shielding wall could also generate unwanted electrostatic forces on the yoke that might reduce the overall driving force. Furthermore, building a thin vertical wall is not easily conducted in surface micromachining technologies, especially if this wall would have to span over several of the other structural and sacrificial layers. So, what we are looking for is a solution to the cross-talk issue that avoids the above issues (as well as possible) and is ok to manufacture in the available process technologies without too much extra effort.

## 2. Materials and Methods

For the simulation of the electric fields, we encased the structural model of a comb drive actuator pixel pair with a cuboid from which the structural bodies were subtracted. Only the remaining volume has been used in the simulations of the electric fields because these are only present there in the air surrounding the metallic structure. This resulted in a FEM mesh of 6.7 million quadratic order elements and 9.8 million nodes.

**Figure 2:**
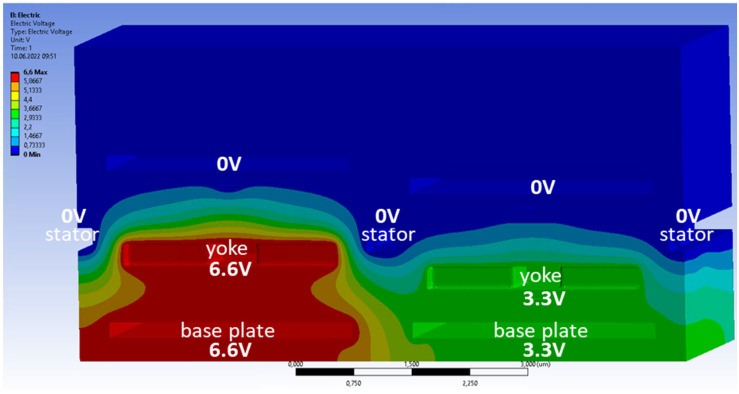
Simulation model of the comb-drive actuator for the cross-talk simulations. The indicated voltages are applied at the interfaces of the labeled structures with the surrounding air. The structural domain is then taken out, and only the electric fields in the surrounding air space are simulated.

To investigate the electrostatic cross-talk, two pixels were simulated that share their long edge with each other. The faces on the outside of this two-pixel cuboid have reflective boundary conditions applied. This means that the electric field vectors are normal to those faces. The top surface is set to zero potential, as are the mirrors, to avoid substantial electric fields above the mirrors. In Figure 2, an exemplary cross-section is shown where the electrostatic potentials can be seen.

A voltage difference between the yoke and stator is applied, which gives rise to a piston-type motion of the yoke, and the maximum deflection is reached when the yoke fingers are partly within the stator comb. This variable addressing voltage is applied at the yoke and at the base plate. By doing this, there is no voltage difference underneath the moveable yoke, and, therefore, only a very small downward force exists due to the fringe fields from the neighboring pixels.

In the real device, a CMOS circuit underneath the base plate provides a variable voltage between 0 V and 3.3 V. To improve the sensitivity and shape of the deflection curve, a constant bias voltage of −3.3 V is applied to the stator and mirror. This results in a voltage difference between the yoke and stator that will be in the range of 3.3 V to 6.6 V. In the FEM simulation, all potentials are shifted by +3.3 V so that the stator and mirror are at 0 V, as discussed above, and the voltage at the yoke and base plate is between +3.3 V and +6.6 V, yielding the same range of voltage differences between yoke and stator. Since all voltages are changed by the same amount, the potential differences between different structures stay the same, and, therefore, the electrostatic forces are unchanged.

Due to the non-linear behavior of the electrostatic force with respect to the voltage difference, the deflection due to the applied bias voltage (and address voltage still at its data = low level) is much smaller than the additional change of deflection when the address voltage is increased from to its data = high level. This additional change of deflection is the stroke of the pixel. The maximum stroke needed for a full 2π modulation of the visible light is 350 nm and has to be reached with the above-mentioned address voltage range. The deflection precision needed for the holographic applications is 8-bit; see [33]. This means that the individual deflection positions are only 1.37 nm apart or 0.4% of the maximum target deflection.

The precision of the deflection of a single pixel is influenced by various parameters. For example, a deviation or variation between pixels of the structural dimensions can lead to a deviation from the target deflection. In addition, a deviation from the intended addressing voltage will lead to deflection errors. In this paper, we investigate the influence of the electric field in one pixel on the electric field in the neighboring pixel. This so-called cross-talk will lead to deviations of the deflection from the target deflection. The larger the difference in the address voltage of neighboring pixels, the larger this cross-talk influence will be.

For our investigation, we compared the two extreme deflection cases. First, we simulated two pixels where both are in the position of the maximum deflection with the maximum address voltage applied. In the second step, one pixel was left in this position, and the neighboring pixel was set in the position of the minimum deflection with the minimum address voltage applied. The pixel that was left in the high position was then evaluated. A similar procedure could be performed for the pixel in the minimum deflection position, but by running a few examples, we found that the maximum deflection position is more sensitive. The relative change of the force was comparable, but due to the lower absolute value of the force needed for the bottom position the same relative force error then results in a much smaller position error for the bottom position.

More precisely, the electrostatic force acting onto the yoke surface was evaluated in both cases and the change of this force is caused by the change of position and address voltage of the neighboring pixel. This change of force is the cross-talk influence for the two-pixel geometry that we investigated. In principle, we would have to correct the position of both pixels due to the changed electrical fields and iterate the force extraction. However, with the small effects we are dealing with, we omitted this kind of iterative process and just calculated the deflection change due to the force change using the mechanical spring constant.

To ensure a meaningful result, we changed the element size of the face meshes at the yoke and stator between 50 nm and 9 nm. Between 9 nm and 15 nm, we did not see a clear change in the cross-talk but rather a fluctuation in the range of 0.1%. The resulting forces also depend, in general, on the mesh structure (changing the finger length leads to a different mesh). Overall, those changes are small (about 0.1% of the extracted force), but since the needed precision of the deflection of the SLM chip is quite strict, this small fluctuation of FEM results is visible in the cross-talk results. We also did not use sharp 90° edges at the yoke and stator fingers but rather smoothed those edges by adding a small rounding radius. This helps to prevent artificially sharp electric field maxima at those edges and numerical instabilities in the FEM extraction of the force z-component.

## 3. Results and Discussion

### 3.1. Cross-Talk of the Initial Actuator Design

**Figure 3:**
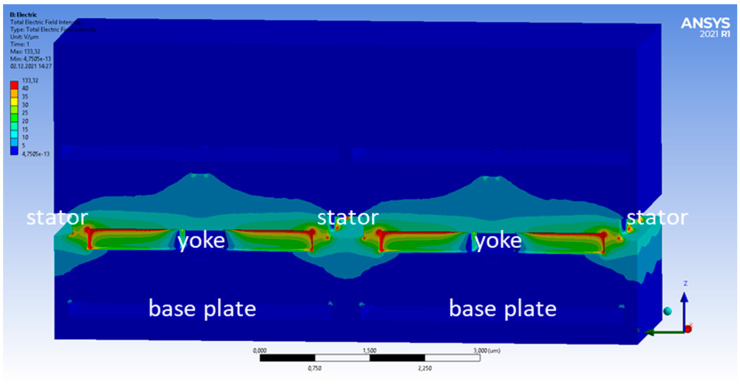
Cross-section through yoke fingers showing the electric field strength for a high/high state.

**Figure 4:**
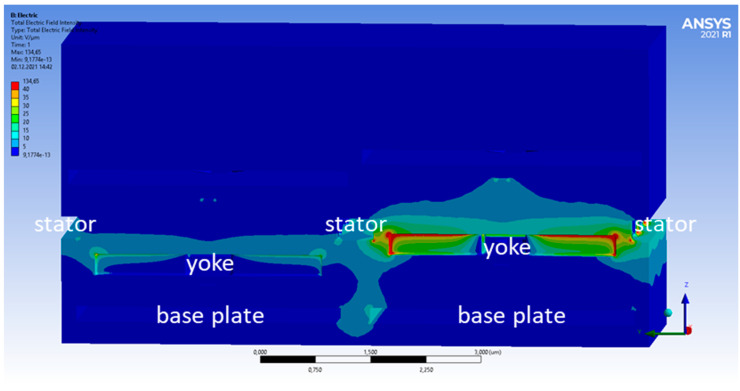
Same cross-section as in Figure 3, but for the high/low deflection case.

In Figure 3 and Figure 4, the electric field intensity is shown for the high/high (Figure 3) and low/high (Figure 4) cases. The color scale is chosen to be non-linear to see the lower amplitude fields better. When both pixels are in the top position, the electric field distribution is symmetric, as expected from the symmetric geometry in this case. In the low/high case, however, the symmetry is broken. One can also see that some electric fields reach from the base plate of the low pixel towards the yoke of the high pixel. This leads to a change in the electric fields reaching the bottom of this yoke. The total force acting on the right yoke is +23.44 nN (high/high) and +23.29 nN (low/high). This change of −0.15 nN arises mostly from the forces at the bottom of the yoke. They increase from −2.66 nN (high/high) to −2.83 nN (low/high). Compared to this, the force at the top of the yoke changes minimally and is +26.10 nN (high/high) and +26.12 nN (low/high), respectively. The change of the total force of 0.15 nN sounds small but still corresponds to a cross-talk of 0.65%.

The final mechanical spring stiffness that will be used will be in the range of 40–50 nN/µm [34]. Using this, we can calculate the difference in deflection a 0.65% cross-talk would cause. At the lower end of the spring stiffness range, we get a deflection difference of 3.75 nm, and for 50 nN/µm, the difference is 3.0 nm.

This variation of the deflection is larger than twice the step size of the individual deflection positions that are to be addressed and, therefore, is too large. Furthermore, in the case investigated here, the cross-talk arises from only one shared border. One can imagine that if the other neighboring pixel with a shared long edge were also in the low state, the cross-talk would be increased by a factor of two. In addition, there may be some more cross-talk from the neighbors at the short sides of the pixel, but this is not simulated, yet.

### 3.2. Design Variations and Their Cross-Talk

**Figure 5:**
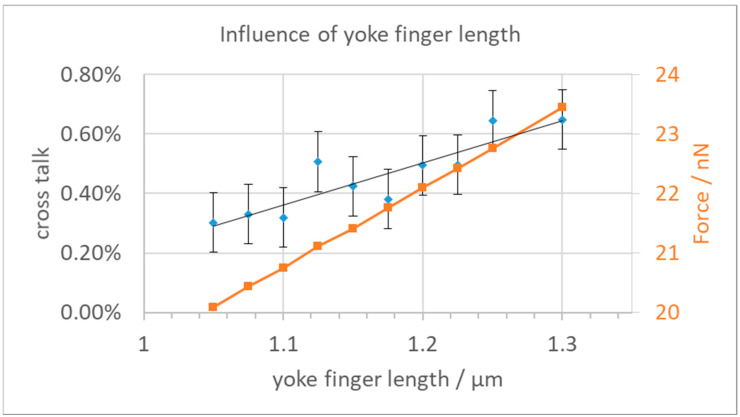
Cross-talk and electrostatic force vs. yoke finger length.

We then investigated various options to reduce the cross-talk while also keeping in mind avoiding too large field strengths and the feasibility of production using surface micromachining technologies, as discussed above. In the first attempt, we reduced the length of the yoke fingers to increase the distance between neighboring yokes and base plates. The cross-talk results are shown in Figure 5, together with the resulting force in the z-direction. As one can see, a reduction of the finger length leads to a small reduction of the cross-talk but also to a substantial reduction of the available force for the deflection of the yoke. The error bars on the cross-talk data points indicate the influence of varying FEM meshes. We found that the resulting forces fluctuate a bit depending on the mesh size at the yoke. The achieved reduction of the cross-talk from 0.65% to about 0.25% is not enough for the needed deflection precision when considering the other neighbors, while the loss in actuator force is already quite painful. This reduction of the actuator force would need softer mechanical hinges, which could only be achieved by reducing the already small dimensions of the two hinges even further.

Increasing the distance between yokes and neighbor base plates can also be achieved by reducing the width of the base plate. We simulated widths from 3.6 µm down to 3.2 µm. However, there was no substantial change in the cross-talk. It remained at a high level of around 0.6%. We think that while a narrower neighbor base plate does have a larger distance from the analyzed yoke, this is shielded to a lesser extent by its own base plate, which is, of course, also narrower in this case.

**Figure 6:**
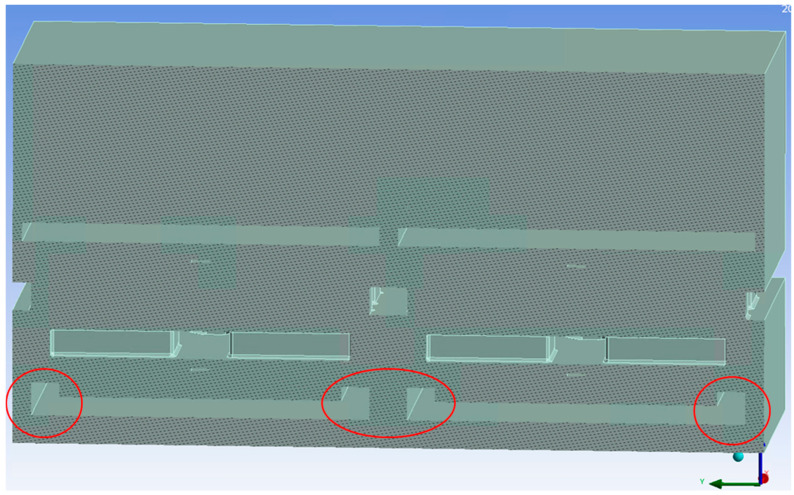
Changed geometry with additional 150 nm tall side walls/rims at the base plate (marked by red circles).

Next, we investigated options to reduce the gap between the yoke and base plate in the hope that this way, the electrostatic influence of the neighbor would penetrate less deeply into the analyzed pixel. One option would be to make an elevated rim at the outer edge of the base plate. However, there the height is limited by the fabrication position of the yoke. In Figure 6, the changed geometry is shown with an additional height of the rim of 150 nm above the base plate (total height above the base plate bottom: 350 nm).

**Figure 7:**
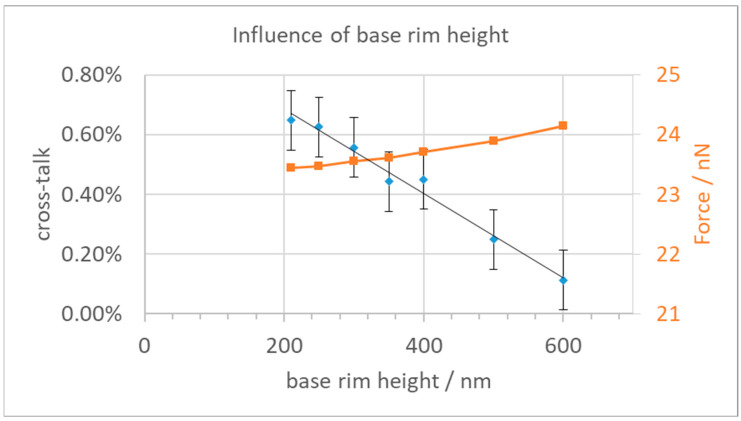
Cross-talk and force vs. height of the base plate rim. Shown is the height above base plate bottom; the base plate center part is 200 nm thick. Used finger length is 1.3 µm.

Increasing the height of this base plate rim did reduce the cross-talk, as shown in Figure 7. Furthermore, the force available for actuation also increased by a small amount. However, for heights of 400 nm and more, the distance between the rim and the yoke fingers becomes too small to be reliably produced; they are just shown here for completeness. Although the rim reduces the cross-talk, if we limit the height to 400 nm, the cross-talk stays above 0.40% for a single neighbor and is still too large for the application.

**Figure 8:**
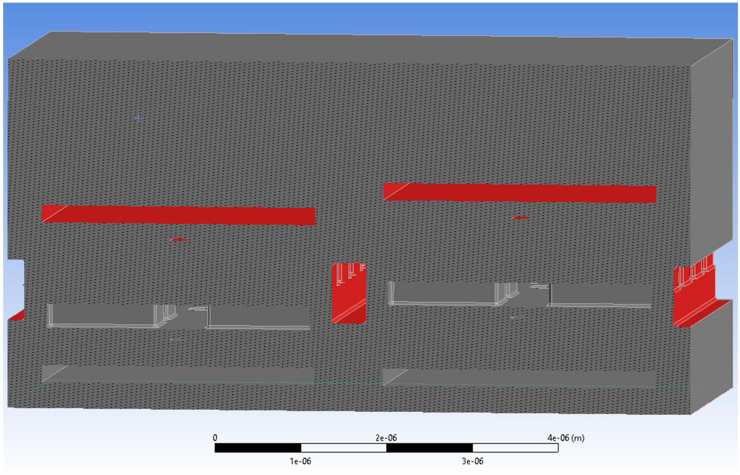
Cross-section through the yoke finger to show the additional stator ‘keel’ (here 400 nm) in between the pixels. Highlighted are the stator, the top hinge, and the two mirror plates.

Another screening option is to add a groove along the pixel edge prior to the deposition of the stator material. This leads to a stator ‘keel’, a screen wall similar to what is discussed above, but with limited height due to manufacturing constraints. Still, some additional shielding material between the two neighboring pixels would be possible with moderate extra effort. We investigated depths of 100 nm to 400 nm for this groove. In Figure 8, the geometry is shown for a 400 nm deep groove.

**Figure 9:**
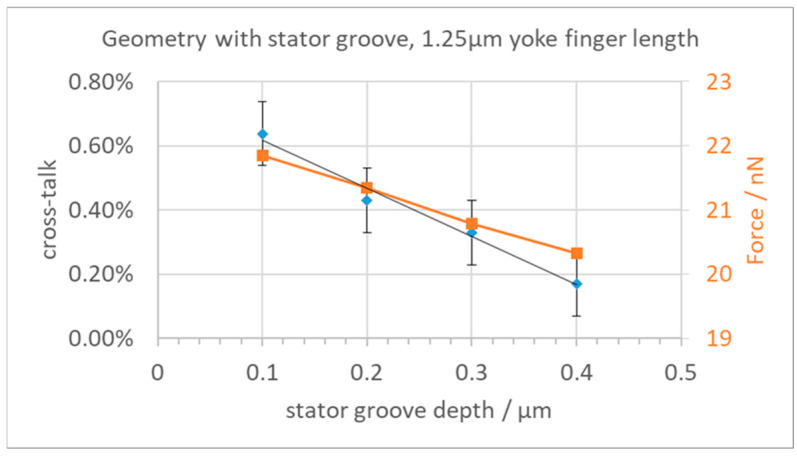
Cross-talk and force vs. stator keel depth.

The cross-talk and resulting total force were investigated for a yoke finger length of 1.25 µm (Figure 9). As can be seen, increasing the keel depth reduces the cross-talk down to 0.2%. However, the electrostatic force is also reduced to 20.3 nN for the deepest stator keel.

**Figure 10:**
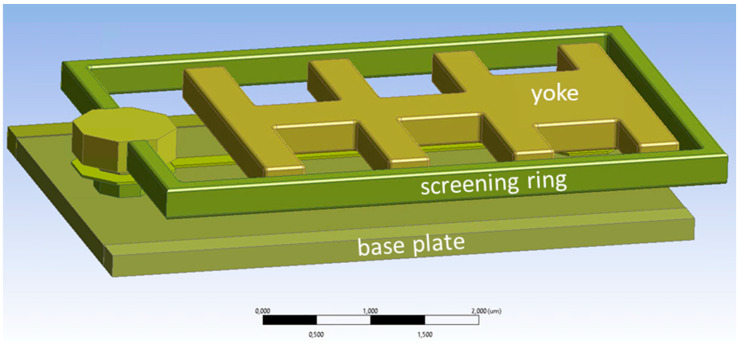
Geometry of the screening ring in yoke layer. Shown are only the layers up this yoke layer.

The final option to reduce the cross-talk is to implement a screening ring fabricated together with and at the same height as the yoke. This also means that no additional layer has to be introduced during the MEMS fabrication; only the mask for the yoke layer has to be adjusted. The structural geometry is shown in Figure 10, the ring has a thickness of 300 nm, like the yoke, and the width of the ring is 200 nm. Obviously, the fingers have to be slightly shorter in this design compared to the initial one to make room for the shield ring.

**Figure 11:**
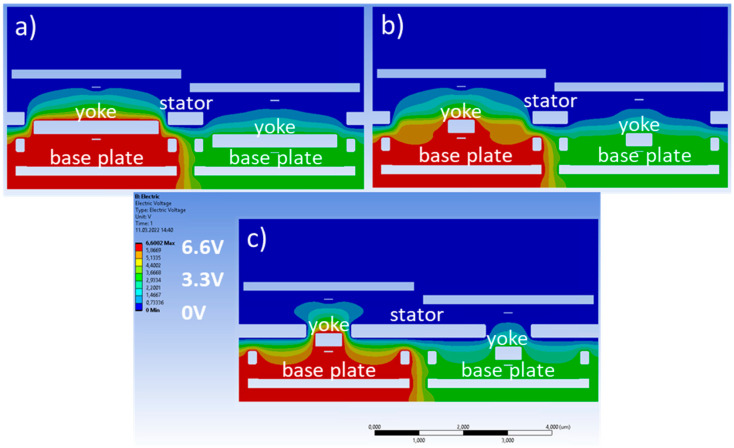
Cross-sectional voltage distribution of the geometry with screening ring at three different lateral positions. (**a**) Cross-section through a yoke finger, (**b**) cross-section through the lateral gap between yoke and stator finger, and (**c**) cross-section through a stator finger.

**Figure 12:**
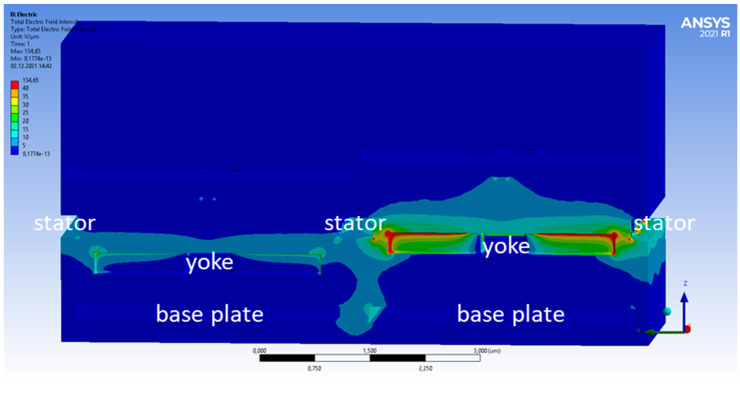
Cross-section through yoke fingers showing the electric field strength with screening ring, as shown in Figure 10.

The shielding effect can be seen quite well when looking at the voltage distribution shown in Figure 11 and at the electric field distribution shown in Figure 12. The ring around the yoke confines the fields to be mostly between the stator and the yoke. One can also see that the field strength at the bottom of the yoke is much lower now. The electrostatic force there is 1.1 nN in both cases (high/high and low/high).

**Figure 13:**
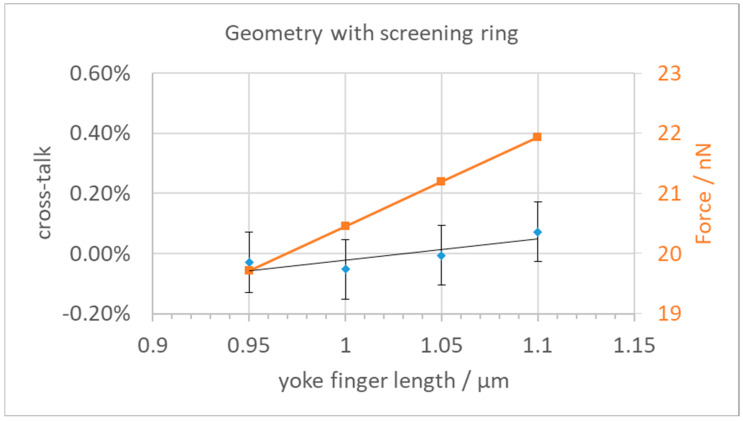
Cross-talk and electrostatic force vs. yoke finger length for the geometry with screening ring.

The calculated cross-talk of around 0.0% is lower than what we expect as a mesh-induced variation (Figure 13). We also see that the available force increased by about 5% when comparing the same finger length with and without a shield ring due to the reduced field strength at the bottom of the yoke. For 1.1 µm finger length, we now get 21.9 nN, whereas, without a ring, it was 20.7 nN. Compared to 23.4 nN for the initial design (see Figure 5), the loss in actuator force is only about half.

**Figure 14:**
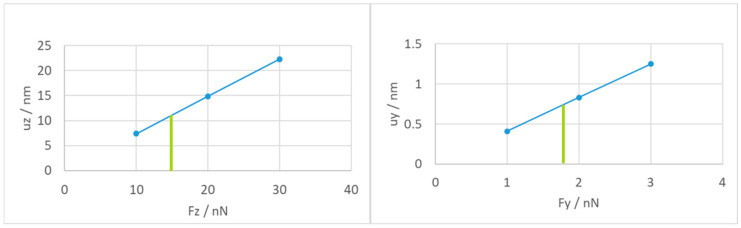
Structural stability of the screening ring. Forces were applied in z-direction (**left**) and lateral in y-direction (**right**), respectively. The green bars indicate the electrostatic force acting on the ring.

We also investigated the mechanical stability of this screening ring. When the yoke is in the top position, the voltage difference between the ring and the stator is largest at 6.6 V. There, the electrostatic force acting on the screening ring is 15 nN towards the stator above (Fz) and about 1.8 nN lateral towards the neighboring ring at 3.3 V. The mechanical stability was simulated by applying a force in the z-direction at the top of the screening ring (only the short segment opposite its post), which will over-estimate the resulting deformation due to the too-large lever arm of the force with respect to the fixed post. The deflection in the z-direction is shown in Figure 14 (left). As can be seen with a force of 15 nN, we expect a deflection of the short edge of the ring of 10 nm.

The lateral stability was investigated by applying a force in the y-direction at the last segment of the ring, which again will over-estimate the resulting deformation. The deflections in this case are also shown in Figure 14 (right). The lateral deformation is below 1 nm and can, therefore, be neglected. The upwards bending of 10 nm is also much lower than the vertical distance of 400 nm between yoke top and stator bottom and is therefore also not a problem.

## 4. Conclusions

We investigated the electrostatic cross-talk of two neighboring comb drive actuators in a micro mirror array designed for computer-generated holography by using FEM simulations of the electrostatic fields and extracting the resulting forces. Structural improvements were needed because the initial cross-talk was larger than the desired deflection precision of 0.4% of the total stroke (8-bit resolution). Various actuator design modifications have been analyzed for their potential to reduce cross-talk. Very small gaps between mechanical parts at different electrical potential would lead to very large electrostatic field strength posing a risk of electrical breakdown and had to be avoided. The final solution is to add a screening ring in the plane of the actuator yoke, which confines the electric fields very well to within each pixel and reduces the simulated cross-talk to levels below the numerical uncertainties of the FEM simulations and well below the specification. This solution does not require any additional fabrication steps and comes at only a quite small reduction of the available force.

## Figures and Tables

**Table 1:** Key SLM specifications and chip floorplan.

**Parameter**	**Value**	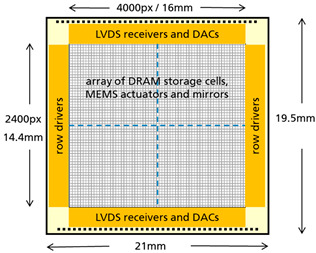
pixel count	4000 × 2400	
pixel size	4 µm × 6 µm	
frame rate	>1 kHz	
vertical deflection range	0 … 350 nm	
deflection precision	8-bit	
mirror tilt	<0.1°	
pixel addressing voltage	0 … 3.3 V	
power dissipation	<2.5 W	

## Data Availability

The original contributions presented in the study are included in the article. Further inquiries can be directed to the corresponding author.
